# Screening of Alginate Lyase-Producing Bacteria and Optimization of Media Compositions for Extracellular Alginate Lyase Production

**DOI:** 10.6091/.21.1.48

**Published:** 2017-01

**Authors:** Hadis Tavafi, Ahya A Abdi- Ali, Parinaz Ghadam, Sara Gharavi

**Affiliations:** 1 Department of Microbiology, Faculty of Biological Sciences, Alzahra University, Tehran, Iran; 2 Department of Biotechnology, Faculty of Biological Sciences, Alzahra University, Tehran, Iran

**Keywords:** Alginate, Bacillus spp, Alginate lyase, Pseudomonas biofilm

## Abstract

**Background:**

Alginate is a linear polysaccharide consisting of guluronate (polyG) and mannuronate (polyM) subunits.

**Methods:**

In the initial screening of alginate-degrading bacteria from soil, 10 isolates were able to grow on minimal medium containing alginate. The optimization of cell growth and alginate lyase (algL) production was carried out by the addition of 0.8% alginate and 0.2-0.3 M NaCl to the culture medium. Of 10 isolates, one was selected based on its fast growth rate on minimal 9 medium containing 0.4% sodium alginate. The selected bacterium, identified based on morphological and biochemical characteristics, as well as 16S rDNA sequence data, was confirmed to be an isolate belonging to the genus *Bacillus* and designated as *Bacillus* sp. TAG8.

**Results:**

The results showed the ability of *Bacillus* sp. TAG8 in utilizing alginate as a sole carbon source. *Bacillus* sp. TAG8 growth and algL production were augmented with an increase in sodium alginate concentration and also by the addition of 0.2-0.3 M NaCl. Molecular analysis of TAG8 *algL* gene showed 99% sequence identity with algL of *Pseudomonas* *aeruginosa* PAO1. The algL produced by *Bacillus* sp. TAG8 cleaved both polyM and polyG blocks in alginate molecule, as well as acetylated alginate residues, confirming the bifunctionality of the isolated lyase.

**Conclusion:**

The identification of novel *algL* genes from microbial communities constitutes a new approach for exploring lyases with specific activity against bacterial alginates and may thus contribute to the eradication of persistent biofilms from clinical samples.

## INTRODUCTION

Alginate, a linear unbranched hetero-polysaccharide, is composed of (1–4)-linked β-D-mannuronate (M) and α-L-guluronate (G), which are arranged by glycosidic bonds as a polyM block, a polyG block, and a random polyMG block[1,^2^]. The relative amount and distribution of these two residues vary with species and growth conditions[3].

Alginate is produced by brown algae and some bacteria belonging to the genera *Azotobacter*, as well as several species of *Pseudomonas*[4] and is also found in acetylated and non-acetylated forms. Alginate produced by brown seaweeds is not acetylated but some of the M residues in bacterial alginates may be O-acetylated on the 2 and/or 3 positions of D-mannuronate[5]. The acetylated form of alginate is synthesized by certain bacteria, such as mucoid cells of *Pseudomonas* *aeruginosa* and *Azotobacter vinelandii. P. aeruginosa* causes serious chronic pulmonary infections in the lungs of patients with cystic fibrosis, and the alginate produced by bacterial cells seems to play a crucial role in the adherence of the bacterium to target cells and biofilm formation[6]. Due to the contribution of alginate to the formation of mucoid biofilm structure, its function in bacterial virulence, and its role in the persistent nature of lung infections, alginate has long been considered as an important therapeutic target[7]. Biofilms prevent effective antibiotics treatment and decrease the uptake and the early bactericidal effect of aminoglycoside antibiotics, thus making treatment of biofilm-dependent bacterial infectious diseases difficult. This feature makes the alginate as an important virulence factor in infections[8]. *P. aeruginosa* has been used as a model in genetic studies of bacterial alginate biosynthesis since all *P. aeruginosa* strains have been shown to carry the genes that encode the regulatory and biosynthetic machinery for alginate production[9].

Alginates are enzymatically depolymerized by alginate lyases (algLs), which catalyze the degradation of the glycosidic bonds between D-mannuronate and L-guluronate by β-elimination and generate a product containing 4-deoxy-L-erythro-hex-4-enopyrano-syluronic acid as the non-reducing terminal end[10]. algLs are classified into three groups by their substrate specificity: the first type is specific toward G block (EC4.2.2.11), the second type is specific toward M block (EC4.2.2.3), and the third type is bifunctional for G and M blocks. Those alginates specific to G or M blocks are called monofunctional algLs, while those specific to MG blocks are called bifunctional algLs[11].

algLs are found both in non-alginate synthesizing and alginate synthesizing organisms. In the non-alginate synthesizing organisms, algLs play important roles in the absorption of alginate as a carbon source[12]. In this case, microorganisms are dependent on the depolymerization activity of algL. In general, microorganisms that grow on alginate secrete extracellular algLs to degrade alginate and then transport the degraded product into the cell for assimilation via cellular metabolism[13]. Although some strains express algL activities constitutively, most bacterial extracellular algLs exhibit their activities only when the host cells are cultivated in the presence of alginate[11]. algLs are produced by a number of microorganisms, including marine algae, marine molluscs, fungi, bacteria, bacteriophages, and viruses[14]. Gram-positive bacteria such as *Bacillus circulans* and Gram-negative bacteria like *Azotobacter vinelandii, Klebsiella aerogenes, K. pneumonia, Pseudomonas maltophilia*, *P. putida*, and *P. 1aeruginosa* have also been reported to produce algLs[15]. Although decomposition of alginate by microorganisms appears to be carried out almost entirely by eubacteria, few Gram-positive bacteria have been identified as the producers of algL[16]. Among alginate biofilm-producing bacteria, *P. aeruginosa* is a clinically important pathogene.. Furthermore, alginate biofilm is a crucial virulence factor in lung infections by this bacterium. algL removes exopolysaccharide from the surface of mucoid *Pseudomonal* cells *in vivo* and *in vitro* and inhibits the adherence of the mucoid strain of *P. aeruginosa*, as well as promotes the diffusion of aminoglycosides through the extracellular polysaccharide of mucoid *P. aeruginosa*[17]. Therefore, the lyase can be used as an adjuvant therapeutic agent for the treatment of infection by mucoid strains of *P. aeruginosa*. algL has also a potential application for enhancing the bacteriocidal effects of antibiotics against mucoid *P. aeruginosa* in biofilms[18].

Considering the enzymatic treatment of infections caused by biofilm-forming bacteria, a search was carried out for a new algL from soil bacteria[19]. Moreover, genetic studies on algL-producing micro-organisms have revealed that the *algL* genes are clustered with other alginate biosynthetic gene loci[20]. Hence, in the present study and based on the presence of *algL* gene, an algL-producing bacterium named *Bacillus* sp. TAG8 was isolated from soil. In order to increase the production yield of algL by the selected isolate, cell growth and conditions for enzyme production were optimized on the basis of various concentrations of alginate and NaCl. The extracellular bifunctional alginate lyase produced by *Bacillus* sp. TAG8 was analyzed based on its interaction with non-acetylated and acetylated alginate.

## MATERIALS AND METHODS

### Screening of alginate lyase-producing bacteria

To isolate alginate-degrading bacteria, 1.0% sodium alginate solution was poured over specified 100 cm^2^ areas of soil once a week for three months[19]. In addition, another area of the soil was sampled directly (without alginate solution). In both methods, about 1 g of the soil samples was mixed with 10 mL sterilized saline solution and shaked. Then the supernatant fluid was filtered and cultured on agar medium containing 0.5% alginate. Medium for the initial isolation of bacteria consisted of 0.5% sodium alginate, 1.0% peptone, 0.5% yeast extract, 0.5% NaCl, and 1.5% agar (pH 7.0). Bacteria were incubated at 37°C for 48 h. To confirm the capability of alginate utilization by the isolated bacteria, colonies from both screening methods were cultured on minimal 9 (M9) agar medium containing 0.2, 0.4, 0.6, and 0.8% (w/v) sodium alginate. The selection criteria of the colonies were rapid growth (in 24 h) in the lowest concentration of sodium alginate. A colony with the fastest growth rate on the M9 medium containing 0.4% sodium alginate was selected for further studies. Isolated bacterium was cultured in M9 medium and Luria-Bertani (LB) broth to investigate the effects of alginate and NaCl on the cell growth and alginate lyase production.

### Biochemical and molecular characterization of the isolated strain

The selected bacterium was isolated by standard dilution plating methods on M9 agar medium consisting of 0.4 to 0.8% (w/v) alginate at 37°C. For identification of the isolate, Gram staining procedure and the following biochemical tests were carried out: oxidase, catalase, Voges-Proskauer, indol and H_2_S formation, hydrolysis of starch, gelatin, esculin, phenyl alanine and casein, nitrate reduction and acid formation of glucose, sucrose, lactose, and hemolysis test[21].

For molecular identification, the isolate was incubated in a LB broth at 37°C overnight. The cells were harvested by centrifugation and the genomic DNA was extracted using PCR template purification kit (Kiagen, Iran). The amplification of 16S rDNA fragments was performed by thermal cycles as: an initial denaturation at 95°C for 10 min, followed by 35 cycles of denaturation at 94°C for 60 s, annealing at 58°C for 30 s, extension at 72°C for 60 s, and a final extension at 72°C for 10 min. Primers used are shown in Table 1.

**Table 1:** Primers used for determining nucleotide sequences of 16S rDNA and the *algL* gene from *Bacillus* sp. TAG8 genomic DNA.

Primer	Sequence (5’ to 3’)	Use
27F	AGAGTTTGATCCTGGCTCAG	Amplification of 16S rDNA gene
492R	GGCTACCTTGTTACGACTT	
AlgL F	ATGAAAACGTCCCACCTGATCCG	Amplification of alginate lyase gene
AlgL R	TCAACTTCCCCCTTCGCGGC	

Due to the importance of finding new algLs that could be effective on *P. aeruginosa* biofilms, the criteria for bacteria selection were based on the ability to degrade alginate as the sole carbon source, as well as to carry the *algL* gene. The sequence for this gene (NCBI Reference Sequence: NC_002516.2) was used as a template to design forward and reverse primers (Table 1). PCR amplification was performed using extracted genomic DNA as a template with the following program: initial denaturation at 94°C for 5 min, followed by 35 cycles of denaturation at 94°C for 30 s, 63°C for 50 s, 72°C for 95 s, and a final extension at 72°C for 10 min. PCR products were then extracted from the gel by Gel/PCR DNA Isolation System kit (Viogene, Taiwan) and sequenced (Macrogen, Korea). The 16 s rDNA sequences and *algL* gene were aligned with multiple sequences from databases in NCBI Database using Blastn. The phylogenetic tree was constructed using neighbor-joining algorithms (Mega 5).

### Effect of alginate and NaCl concentrations on cell growth and alginate lyase activity

In order to evaluate the effect of sodium alginate and NaCl on cell growth and extracellular lyase activity, *Bacillus* sp. TAG8 was cultured in M9 and LB broth containing 0.2 to 0.8% sodium alginate as well as in M9 and LB broth containing 0.1 to 0.5 M NaCl (3 replicates per treatment) at 180 rpm at 37°C for 18 h. During incubation (3 hours), samples were withdrawn periodically, and the optical density in each treatment was measured at 600 nm to assess the growth rate. Bacterial growth curve was subsequently plotted. In addition to assaying the enzyme activity from the treated media, the cell pellets were centrifuged at 4000 ×g at 4°C for 10 min to obtain supernatant (as a source of enzyme) to estimate the extracellular algL activity.

### Extracellular alginate lyase activity assay

For measurement of the enzymatic activity of algL, 0.2 ml of supernatant was added to 1 ml of 1.0 M Tris- HCl buffer (pH 8.3) containing 0.1% sodium alginate as substrate and incubated at 30°C for 5 min. Next, the reaction was stopped by heating in boiling water for 5 min. The enzyme activity was assayed by measuring the increase in absorbance at 235 nm, which is a result of double bond formation at the non-reducing end by β-elimination reaction. Furthermore, deoxy sugar formation in the supernatant was determined by tiobarbitoric acid (TBA) method at 548 nm[4,15]. The results are represented as enzyme units, where one unit of enzyme activity is defined as the amount of enzyme that generates 1 nmol of β-formyl-pyruvate per min per ml at 37°C[15].

### Substrate specificity of the enzyme

In order to study the substrate specificity of the enzyme isolated from *Bacillus* sp. TAG8, polyM and polyG blocks of alginate were used as substrates. The blocks were prepared by mild acid hydrolysis of alginate as described by Kashima and Imai[22], and the chemical composition of the blocks was determined by H-NMR analysis. Finally, the optimal enzymatic activity was measured using the TBA method[23] in optimal concentrations of 0.3 M NaCl and 0.8% sodium alginate for 12 h of incubation time.

### Statistical analysis

Optimum conditions for cell growth (absorbance at 600 nm) and algL production assay (absorbance at 235 and/or 548 nm) were established, and the results were analyzed statistically. Significant differences were determined by one-way analysis of variance (ANOVA one-way) with pairwise comparisons using Tukey’s method. A *P* value <0.05 was considered statistically significant. Statistical analysis was performed using the Prism5 software.

## RESULTS AND DISCUSSION

For the isolation of algL-producing bacterium, an initial screening was performed on M9 agar plate containing 0.4% (w/v) sodium alginate as the sole carbon source. After incubation at 37°C for 48 h, 10 colonies appeared on the agar plate with algL activity. Interestingly, no difference was observed in the diversity of the isolated bacteria from sprayed soil samples with or without sodium alginate solution. However, Nakagawa *et al*.[19] have reported that treatment with sodium alginate solution is an effective method for the isolation of lyase-producing microorganisms. Most of the colonies were cream-colored, pale yellow, and wrinkled but some of them were flat. These isolates showed a better growth on plates containing 0.6 and 0.8% alginate; however, no growth was observed in the medium containing 0.2% and 0% sodium alginate. Based on the relative size of colonies on M9 agar medium containing 0.4% (w/v) sodium alginate, one colony with the fastest growth rate (in 24-h incubation) was selected for further study and analyzed by PCR for the desired *algL* gene. *Bacillus* sp. TAG8 colonies were cream-colored and classified as Gram-positive bacterium with characterized biochemical properties (Table 2). The growth temperature range was 27-37°C, and the optimal growth temperature in LB or M9 broth media was 37°C. Other isolated colonies showed lower growth rates at 37°C, as compared to *Bacillus* sp. TAG8. Based on the biochemical properties, *Bacillus* sp. TAG8 isolate showed >79% similarity to *Bacillus* species (http://www.microrao.com/identify.html and http://www.tgw1916.net/bacteria_abis.html). For the precise identification of *Bacillus* sp. TAG8, the 16S rDNA sequence was determined and analyzed using Blastn. Homology studies revealed that 16S rDNA gene of the strain TAG8 showed sequence identities of 99% with *Bacillus atrophaeus* strain NXUSASNFB001, Bacterium NS7, *Bacillus atrophaeus* strain AECSB18, *Bacillus methyltrophicus* strain LH-T8, *Bacillus pumilus* strain 3L0-10E, Basillus sp. SDHR2 and 98% with the strain Bacillus sp. 13422.

**Table 2:** Biochemical properties of the *Bacillus* sp. TAG8

Properties	*Bacillus* sp. TAG8
Gram stain	+
Shape	Rods
Spore	+
Motility	+
Pigmentation	cream/dark cream
	
Enzyme activity of:	
Oxidase	-
Catalase	+
VP test	+
Indole	-
H_2_S formation	-
	
Hydrolysis of:	
Starch	+
Gelatin	+
Esculin	-
Phenyl alanine	-
Casein	+
Nitrate reduction	+
	
Acid formation of:	
Glucose	+
Sucrose	+
Lactose	-
Hemolysis	+

**Fig. 1:**
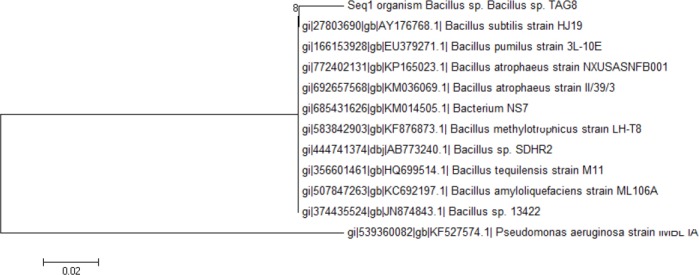
Phylogenetic analysis based on the 16S rDNA sequences from *Bacillus* sp. TAG8 and eleven other bacteria. Neighbour-joining model was employed for the tree construction, and bootstrap values were obtained with 1000 repetitions. The bar labeled 0.02 indicates 2 base changes per 100 nucleotides.

The phylogenetic tree was constructed using the neighbor-joining method on the basis of the comparative sequence analysis of 16S rDNA (Fig. 1). Based on the evaluation of biochemical, morphological, and physiological characteristics and 16S rDNA sequence analysis, it is concluded that TAG8 belongs to the genus *Bacillus* and is named *Bacillus* *sp*. TAG8. The nucleotide sequence of the 16S rDNA of *Bacillus* sp. TAG8 was submitted to GenBank (accession number KR267304).

**Fig. 2:**
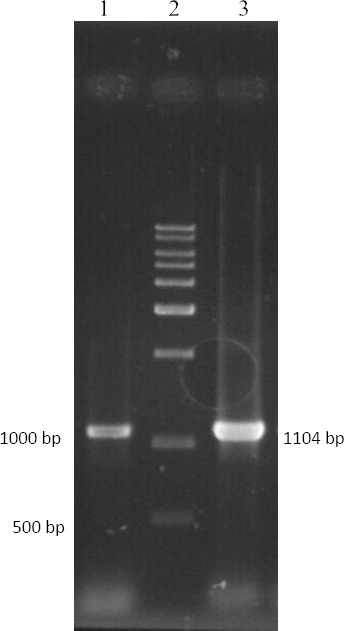
PCR product *algL* gene amplification from PAO1 and TAG8. Lane 1, *Bacillus* sp. TAG8; Lane 2, molecular weight standard (10 kbp, Fermentase, Lithuania); Lane 3, PAO1 strain.

One important aspect of this study is to ultimately find an algL that can be effective in the elimination of *P. aeruginosa* biofilm. In this study, the presence of *algL* gene was important in the screening of bacteria. The primers were designed to facilitate the screening (Table 1). *Bacillus* sp. TAG8 genomic DNA was used as template for PCR amplification of the *algL* gene. PCR product of the gene was ~1104 bp (Fig. 2). Furthermore, the subsequent blast analysis on *algL* gene showed that the closest relative of the *Bacillus* sp. TAG8 was *P. aeruginosa* PAO1 (99%). The *algL* sequence of *Bacillus* sp. TAG8 was submitted to GenBank (accession number KR267305).

**Fig. 3:**
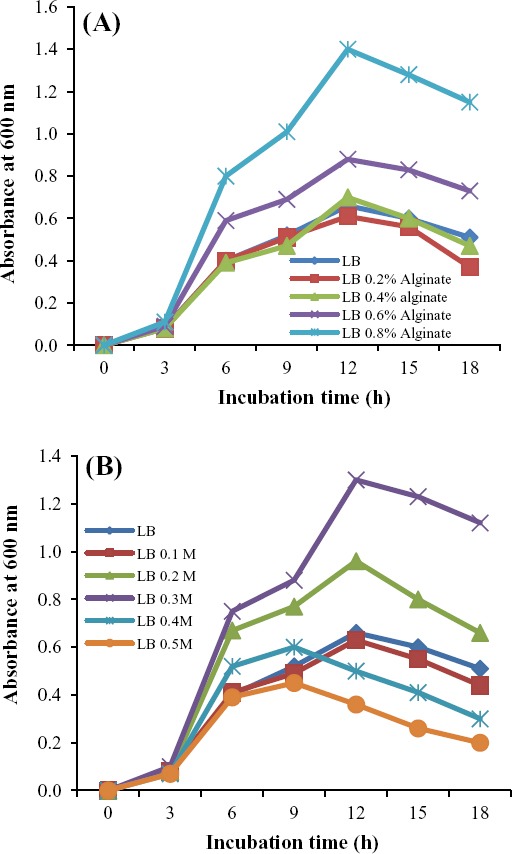
Growth curve of *Bacillus* sp. TAG8 on Luria-Bertani medium containing various concentrations of alginate (A) and various amounts of NaCl (B) at 180 rpm, 18 h incubation time and 37°C.

In order to study the relationship between cell growth and algL production, *Bacillus sp*. TAG8 was cultured on M9 and LB broth medium containing sodium alginate and NaCl. As expected, growth was inhibited when M9 broth medium without carbon source was used. However, growth in the presence of sodium alginate clearly indicates that *Bacillus* sp. TAG8 is capable of using alginate as the sole carbon source. Faster growth rates and the appearance of colonies were observed at low concentrations of alginate (0.4%). Park *et al*.[24] isolated strain MJ-3 and reported that the growth rate of the isolate is significant in M9 medium containing 0.8% alginate. However, the growth rates of the isolates were increased with higher sodium alginate concentrations in LB broth (Fig. 3A). Therefore, this medium was used for the optimization of algL production.

In addition to the study of various alginate concentrations on bacterial growth, the effect of different concentrations of NaCl (0.1-0.5 M), as a supplement, was also investigated. The results showed that the concentrations of 0.2 and 0.3 M NaCl improved the bacterial growth compared to 0.4 and 0.5 M NaCl; however, higher NaCl concentrations did not increase the cell growth rate (Fig. 3B).

algL activity has been determined by various methods[1,15,24,25]. This enzyme depolymerizes alginate by β-elimination reaction to produce 4 deoxy-erytro-hex-4-enopyranuronosyl groups at non-reducing end[1]. Among assays to measure extracellular algL activity, ultraviolet absorption method, in which the absorbance at 235 nm is measured for the supernatant[24], is prevalent. Muramatsu[26] has reported that for the measurement of the kinetic parameters of enzymatic reaction, this assay has been proven to be both simple and rapid. The absorbance at 235 nm reflects the production of unsaturated uronates[27]. In the current study, in addition to measuring the enzyme activity at 235 nm, an accurate and a quantitative TBA method were also used to measure the enzyme activity.

**Fig. 4:**
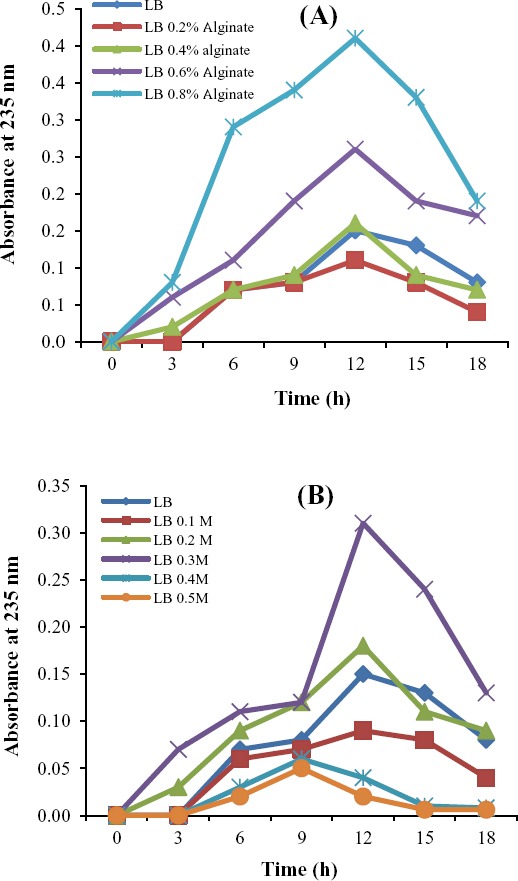
Alginate lyase activity of *Bacillus* sp. TAG8 was grown on LB medium containing different concentrations of alginate (A) and NaCl (B). At 18 hours of incubation at 37°C, the alginate lyase activity was measured based on the increase in absorbance at 235 nm.

As *Bacillus* sp.TAG8, isolated in this study, is an extracellular algL producing bacterium, algL activity was assessed in the supernatant of the bacterial culture containing different concentrations of sodium alginate and NaCl. The results showed that the levels of enzyme activity increased significantly with increase in the alginate concentration (*P*<0.05) (Fig. 4A). However, algL activity did not increase in 0.4 and 0.5 M NaCl concentrations compared to 0.2 and 0.3 M NaCl (Fig. 4B). Eftekhar and Schiller[15] studied the effect of NaCl concentrations on lyase activity of *P. aeruginosa* and concluded that increasing the NaCl concentration can not elevate the algL production yeild.

**Fig. 5:**
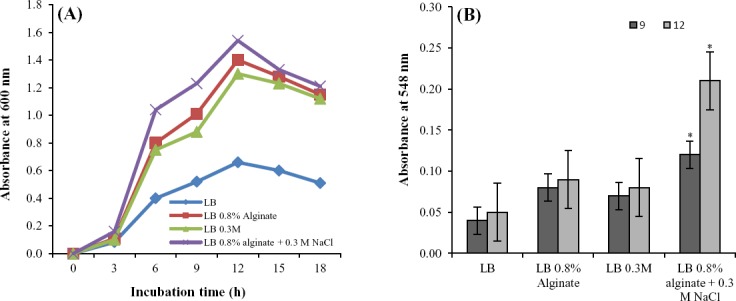
The growth curve of *Bacillus* sp. TAG8 on LB medium containing 0.8% alginate+0.3 M NaCl and LB medium containing 0.8% alginate and 0.3 M NaCl in 18 hours of incubation at 37°C (A) and alginate lyase activities of *Bacillus* sp. TAG8 grown in LB medium containing 0.8% alginate+0.3 M NaCl and LB medium containing 0.8% alginate and 0.3 M NaCl (B). Supernatant of culteres were collected after 9 and 12 hours of incubation, and alginate lyase activity was recorded based on the increase in absorbance at 548 nm by TBA method. ANOVA with pairwise comparison using Tukey’s method was used to compare effect of NaCl and sodium alginate concentrations simultaneously that showed increased alginate lyase activity significantly compared to the control LB and each treatment separately (*P<0.05). Error bars indicate standard errors of mean of 4 experiments.

Effects of sodium alginate and NaCl concentrations on the growth rate of bacteria were studied simultaneously. The results showed that medium containing 0.8% sodium alginate and 0.3 M NaCl stimulated bacterial growth almost 1.2-fold more than when cultures were treated separately, and almost 2.3-fold more than when bacteria were grown in LB medium without alginate (*P*<0.05) (Fig. 5A). These results suggest that *Bacillus* sp.TAG8 is able to use NaCl as a co-factor for the degradation of the alginate in its metabolic pathway. Results also indicate that the simultaneous presence of NaCl and sodium alginate increased the algL activity significantly, as compared to the control LB or individually added salts (*P*<0.05) (Fig. 5B). Furthermore, the enzymatic activity of algL was 10.5 enzymatic units in optimum conditions, demonstrating an almost 2-fold increase as compared to 0.8% alginate and 0.3 M NaCl treatments. Bacterial growth curve analysis showed that the algL activity of *Bacillus* sp.TAG8 continuously increased up to the stationary phase and gradually decreased, which indicates that lyase activity was related to cell growth. Park and coworkers[24] have presented data that supports our results.

**Fig. 6:**
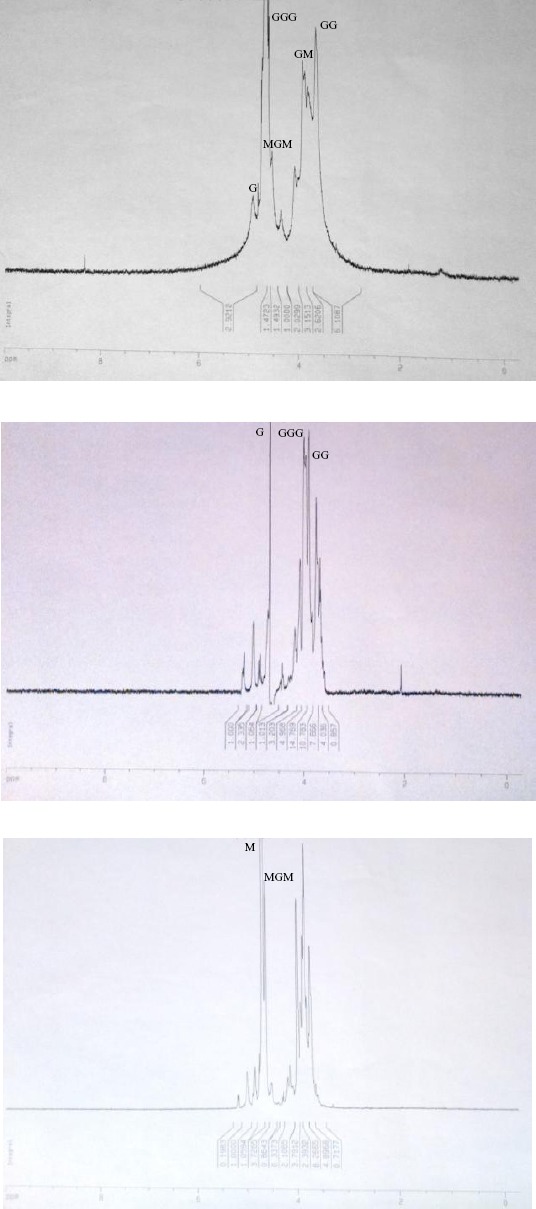
The H-NMR (300 MHz) spectra of solution of (A) sodium alginate, (B) blockG (guluronate), and (C) blockM (mannuronate).

In the current study, lyase activity on different alginate substrates was examined to investigate the substrate specificity of the enzyme. The substrates were prepared by the partial hydrolysis of alginate as described earlier[22]. PolyM and polyG blocks were isolated, and chemical composition of each block was confirmed by H-NMR (Fig. 6). The results of lyase activity by TBA method showed that algL from *Bacillus* sp. TAG8 almost equally degraded polyM and polyG blocks after incubation for 12 h. The lyase activity of TAG8 on polyM and polyG blocks was reported as the optical density of 0.2 and 0.17 at 548 nm, respectively. One unit is defined as the release of 1 nmol β-formyl-pyruvate per minute and 10 nmol β-formyl-pyruvate corresponds to an absorption of 0.29 at a wavelength of 548 nm[15]. Therefore, the enzyme produced by *Bacillus* sp. TAG8 has been shown to be a bifunctional algL. In this context, Hansen and coworkers[16] identified strain *B. circulans*, which specifically degraded polyM alginate. Nakagawa *et al*.[19] also identified *Bacillus* sp. strain ATB-1015, which had substrate specificity for both the polyG and polyM blocks in the alginate. Moreover, *Bacillus* sp. TAG8 algL showed activity toward the isolated acetylated alginate of mucoid *P. aeruginosa*, indicating that this enzyme is able to degrade acetylated alginate of a pathogen source.

In this study, the growth conditions of isolated alginate-degrading bacterium, *Bacillus* sp. TAG8, from soil was optimized in order to achieve the higher production of algL. As the results indicate, the production and the activity of algL enhanced with an increase in growth rate, which indicats that algL production was directly related to the cell growth. Hence, as expected, the production of enzyme was increased in the logarithmic growth phase. We here assessed the roles of alginate and NaCl in enhancing cell growth and algL production. The results demonstrated that the *Bacillus* sp. TAG8 possesses an alginate-degrading system, which is assisted by NaCl as a metabolic co-factor for the enhanced degradation of alginate. Furthermore, the properties of TAG8 and the effect of its algL on acetylated alginate, makes *Bacillus* sp. TAG8 a potential candidate for further studies on the elimination of *P. aeruginosa* biofilm.
